# Visualization methods for statistical analysis of microarray clusters

**DOI:** 10.1186/1471-2105-6-115

**Published:** 2005-05-12

**Authors:** Matthew A Hibbs, Nathaniel C Dirksen, Kai Li, Olga G Troyanskaya

**Affiliations:** 1 Computer Science Department, Princeton University, 35 Olden Street, Princeton, NJ 08544, USA; 2 Lewis-Sigler Institute for Integrative Genomics, Princeton, NJ 08544, USA

## Abstract

**Background:**

The most common method of identifying groups of functionally related genes in microarray data is to apply a clustering algorithm. However, it is impossible to determine which clustering algorithm is most appropriate to apply, and it is difficult to verify the results of any algorithm due to the lack of a gold-standard. Appropriate data visualization tools can aid this analysis process, but existing visualization methods do not specifically address this issue.

**Results:**

We present several visualization techniques that incorporate meaningful statistics that are noise-robust for the purpose of analyzing the results of clustering algorithms on microarray data. This includes a rank-based visualization method that is more robust to noise, a difference display method to aid assessments of cluster quality and detection of outliers, and a projection of high dimensional data into a three dimensional space in order to examine relationships between clusters. Our methods are interactive and are dynamically linked together for comprehensive analysis. Further, our approach applies to both protein and gene expression microarrays, and our architecture is scalable for use on both desktop/laptop screens and large-scale display devices. This methodology is implemented in GeneVAnD (Genomic Visual ANalysis of Datasets) and is available at .

**Conclusion:**

Incorporating relevant statistical information into data visualizations is key for analysis of large biological datasets, particularly because of high levels of noise and the lack of a gold-standard for comparisons. We developed several new visualization techniques and demonstrated their effectiveness for evaluating cluster quality and relationships between clusters.

## Background

Recent high-throughput and whole-genome experimental methods create new challenges in data analysis and visualization. Gene expression and protein microarrays output hundreds of thousands of data points that can be used for prediction of gene function over the entire genome. However, there are serious and fundamental challenges in the analysis of these data. Microarray data contain substantial experimental noise and as our knowledge of biology is incomplete, no perfect gold standard exists for verification of microarray analysis methods.

In order to determine gene/protein relationships and functions from microarray data, methods must be robust to noise and must identify groups of genes that may be functionally related. Statistical methods, such as clustering, attempt to identify data patterns and group genes together based on various distance metrics and algorithms. The lack of a true gold standard makes it impossible to verify the absolute accuracy of any clustering method. Several statistical approaches have been presented for assessing cluster quality [1-4], but these are all either internal validation methods or methods that rely on incomplete external standards such as MIPS [5] or Gene Ontology [6] functional protein classifications. Further, these methods do not address the issue of identifying specific problems within clusters of microarray profiles or assessing the relationships between clusters of genes. Well designed visualization methods are capable of aiding in these tasks by helping to bridge the gap between raw data and the analysis of that data [7]. To perform more comprehensive cluster analysis, statistically integrative, dynamic, noise-robust data visualizations are required to complement purely analytical evaluation methods.

Existing visualization tools do not include methods to statistically and dynamically evaluate clusterings of genes. Several tools can display expression data in various static ways suitable for publication [8] or provide useful dynamic views of tabular data [9], but are not specifically intended for cluster analysis. JavaTreeView [10] and the HierarchicalClusteringExplorer [11] dynamically display hierarchically clustered data for analysis and VxInsight [12] displays the result of a built-in clustering algorithm in an interactive 3D topology, but none are able to display results of other clustering methods for analysis. TreeMap [13] provides an innovative way to visualize hierarchically clustered data as well as data organized in the context of the GO hierarchy, but is not intended for cluster analysis. New tools such as GeneXplorer [14] provide an interactive method for visualization and analysis of microarray data on websites, but do not focus on the task of cluster analysis. Several tools, including the MultiExperimentViewer [15] and Genesis [16], provide multiple methods of performing clustering as well as some visualization methods to analyze the resulting clusters. Commercial tools, such as GeneSpring [17] and SpotFire [18], offer various statistical and visualization tools for general analysis, but neither offer visual methods specific to analyzing the results of clustering algorithms. Therefore, there is a need for a visualization-based methodology designed specifically to statistically and dynamically evaluate clusters produced by the variety of available algorithms and software tools.

Here we present a suite of interactive microarray analysis methods that integrate relevant statistical information into visualizations for the purpose of assessing the quality and relationships of clusters in a noise-robust fashion. Our methodology is general and can be used to analyze the results of most clustering algorithms performed on either protein or gene expression microarray datasets.

## Results and discussion

### Noise robust visualization

**Figure 1:**
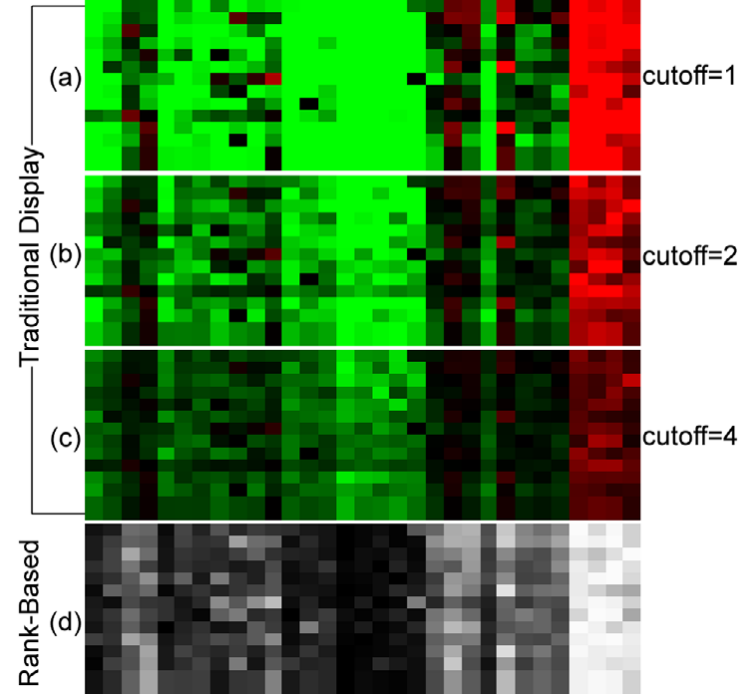
**Example of noise in microarray visualization**. Four views of the same data displayed in different ways. (a-c) show a traditional display using different cutoff values. Note that in (a) variation in the highly over and under expressed regions cannot be seen due to saturation, while in (c) variation in the highly expressed regions can be seen, but variation near zero cannot. (d) uses our rank-based visualization method. In this rank-based view (d), the experiment with the lowest expression for each gene is colored black, the experiment with the highest expression is colored white, and the other experiments interpolate between in grayscale. Using this method, users can see the overall pattern of variation in the data, which makes it clear that heterogeneity in the traditional view is mostly the result of noise. (Data from [26])

Microarray data contain a substantial amount of noise; therefore, visualizations must facilitate tasks like pattern identification and outlier detection in a noise-robust fashion. Microarray data span a rather large and noisy numerical range, so traditional microarray visualizations use a cutoff value that specifies where maximum saturation occurs. While this is necessary in order to see variation around zero, it obscures variation in highly over or under expressed areas (Fig. 1a–c). At a minimum this cutoff value should be dynamically controlled by the user so that they have the ability to see both types of variation. Several currently available tools include this ability, as does our method, but while the ability to change the cutoff value helps to increase dynamic range and decrease the effects of noise in visualizations, it fails to address the entire problem. Traditional visualization methods essentially display the Euclidean distance between gene expression profiles, a measure that is not robust to outliers. Distance metrics more robust to noise, such as a rank-based Spearman correlation coefficient, can be used for numerical analysis of microarray data. We propose a rank-based visualization method to serve as the complement to these noise robust distance metrics (Fig. 1d).

Our method performs a rank transform on each gene by sorting the gene's expression levels, then ranking the experiment for each gene with the lowest expression 0, the next lowest 1, and so on to the highest expression which is ranked *N-1*, where *N *is the number of experiments. Each experiment is then displayed as a grayscale percentage of rank/(*N-1*). In this display, the experiment with lowest expression for each gene is colored black, the experiment with the highest expression is colored white, and the intermediate experiments gradate between them in shades of gray.

**Figure 2:**
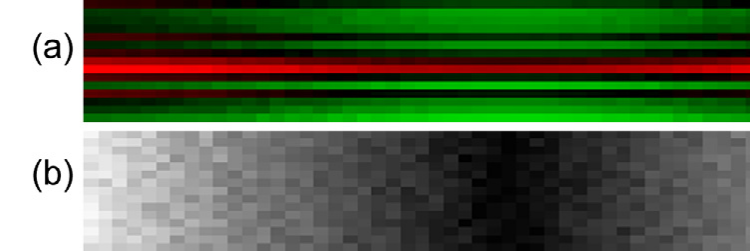
**Rank-based visualization of synthetic data**. Synthetic data displayed (a) traditionally and (b) using our rank-based method. This data was generated by creating a single sinusoidal expression profile and for each gene (row) randomly shifting that profile up or down and introducing small amounts of Gaussian random noise throughout. The result is that the genes generally follow the same shape/trend over experiments, but the shapes are shifted up/down from one another. Traditional view (a) masks the similarity between genes, but their relationship is clear in the rank-based view (b).

In addition to being more robust to noise, this rank-based visualization allows users to easily see patterns of shape/trend that are not apparent in traditional visualizations. Clustering algorithms that use a rank-based distance metric will group together genes based on their pattern of expression which can result in clusters that look very nonuniform when traditionally displayed (Fig. 2). However, in our rank-based visualization it is clear that these genes do belong together because they share expression profiles with the same shape/trend.

**Figure 3:**
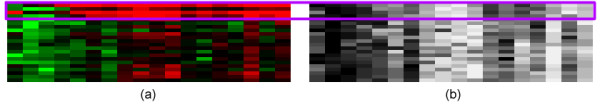
**Rank-based visualization of time series data**. Yeast cell cycle data displayed (a) traditionally and (b) using our rank-based method. In the traditional visualization the top 4 genes (within the purple box) appear to be very different from the rest of the genes in this cluster. However, using the rank-based method it becomes clear that these genes follow the same general pattern of the entire cluster, with initially low expression building up to highest expression in the central time points and then falling to roughly middle values. (Data from [22])

While the example in Fig. 2 is an extreme case, this rank-based visualization approach is useful in a variety of biological settings. For example, in many time series data sets it is useful to observe changes in expression over time in response to some process such as environmental changes, drug introduction, or cell cycle phase. In particular, a group of genes which all rise in expression over a period of samples in a cell cycle experiment, but whose absolute expression levels are not the same will appear heterogeneous when displayed traditionally. However, when displayed using our rank-based method, the pattern of expression is much clearer, which can aid users to identify biologically meaningful trends of expression (Fig. 3). Genes exhibiting a coherent progression of shape/trend over time may be co-regulated. Thus, it is important to identify trends and not just examine similarities of absolute expression level.

### Assessing cluster quality

While multiple statistical methods have been developed for assessing the quality of clusters produced by different algorithms [1,3,4] the most appropriate clustering algorithm choice depends on the dataset, distance metric, and goal of the analysis [2]. Due to the limitations of these methods, it is important to effectively display clustered data in a manner that allows researchers to examine the variation and consistency of the results of different clustering algorithms. We propose two new visualization techniques that can be used to assess overall cluster quality, and also identify individual outliers and other anomalies in the data quickly and efficiently.

**Figure 4:**
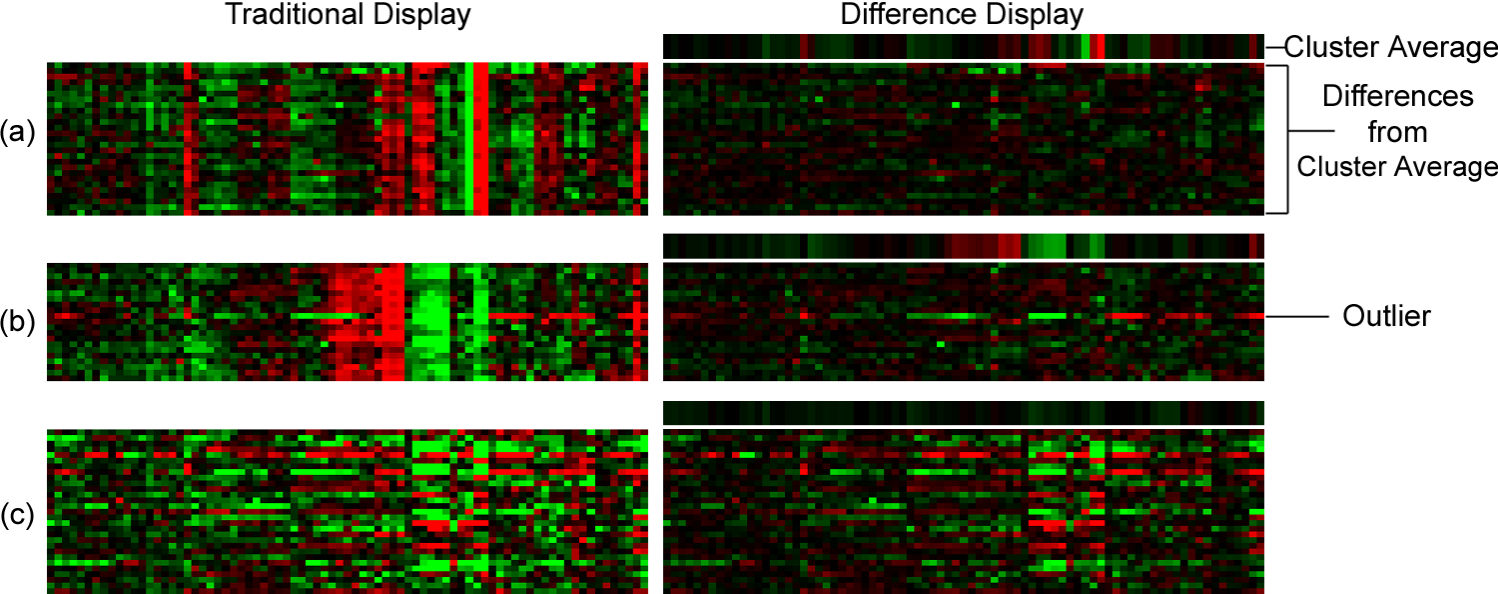
**Difference display visualization**. Three clusters displayed traditionally on the left and in our difference image visualization on the right. In the difference display, the large top bar on each cluster shows the cluster average, each gene is displayed as its difference from that average (green indicates expressed less than the cluster average, red shows more expressed, and black means equally expressed with the cluster average). Cluster (a) is a coherent cluster of genes and appears very dark because of its homogeneity. Cluster (b) is another dark, uniform cluster, but it also contains one randomly inserted gene, which can be easily identified in our difference display. Cluster (c) contains a random selection of genes, and its randomness is clear from the brightness of the difference display. This difference display allows for quick assessment of overall cluster homogeneity and facilitates quick outlier detection. (Data and clusters a & b from [19])

First, to analyze the overall cohesion of each cluster, we developed a "difference display" method. For each cluster, we display the cluster average bar to show the general expression of the cluster as a whole. We calculate the vector of the cluster average  from the vectors of expression profiles of each gene, , for each cluster containing *M *genes with expressions measured over *N *experiments using the standard formula:



Each gene's expression is displayed as a difference, , from the cluster average, :



Thus if a gene is shaded green in an experiment, it is expressed lower than the cluster average for this experiment, and if shaded red it is expressed more in an experiment than the cluster average for that experiment. In this visualization a cluster that is relatively dark is more uniform since the genes are generally close to the average (Fig. 4a). Individual genes that differ from the average more than others will stand out as brighter than their neighbors, which allows for easy visual detection of outliers (Fig. 4b). Thus, this visualization allows researcher to easily identify genes that do not fit well with the cluster's expression profile, and thus may be functionally distinct from the rest of the cluster.

**Figure 5:**
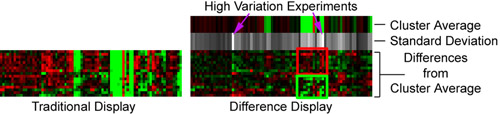
**Experiment variation display**. A cluster displayed traditionally on the left and in our difference image visualization on the right also showing the standard deviation within the cluster for each experiment. Black on the standard deviation bar indicates a standard deviation of zero, while white indicates a higher value. Purple arrows point to several experiments in this cluster that show high variance. In general, the high variance among some experiments may indicate that this cluster is unregulated under those conditions. In this example, we can inspect the differences from the cluster average in the high variance experiments and see that for these conditions the upper group of genes (indicated by a red box) is less under expressed than the lower group of genes (indicated by a green box) which suggests that the cluster could be split into two sub-clusters to reduce this variation. The biological function of these genes is consistent with such a split (see web supplement for details, . Data and cluster from [19])

Second, in addition to assessing overall cluster quality and identifying gene outliers, it is important to look at variation of individual experiments within each cluster. We calculate the standard deviation, *s*, of each experiment, *j*, within a cluster in the normal manner:



Where *M *is the number of genes in the cluster,  is the cluster average for experiment *j*, and *g*_*i*, *j *_is the expression level of gene *i *in experiment *j*. We display the standard deviation of each experiment within the cluster below the cluster average bar. Here black indicates a standard deviation of zero and white indicates higher standard deviations, saturating at a user defined cutoff value. This allows a user to quickly identify high and low variation experiments on a per-cluster basis (Fig. 5). High variation experiments may imply that the genes in this cluster were less related under those particular experimental conditions.

Visualizing clusters in this difference display method allows users to see variations in expression level that may be biologically significant that are not visible in traditional visualization methods. For example, the data shown in Fig. 5 is the glycolysis cluster (2E) from [19]. When viewed traditionally this cluster appears very homogenous and consistent. However, when viewed as a difference from the cluster average, we can observe that in the region of highly under-expressed experiments some genes are more expressed than the average while others are less expressed than average (red and green boxes are shown in this area). This suggests that the cluster could be split into two smaller clusters that would be even more homogenous. In this example 8 of the 9 genes indicated by the red box, but only 3 of the 8 genes indicated by the green box are annotated to glycolysis. The genes in the green box are better categorized as more generally related to alcohol metabolism than to glycolysis in particular (see web supplement to Fig. 5 for details, located at ). Traditional visualization is unable to show this type of biologically meaningful variation in highly over or under expressed regions.

### Assessing cluster relationships

**Figure 6:**
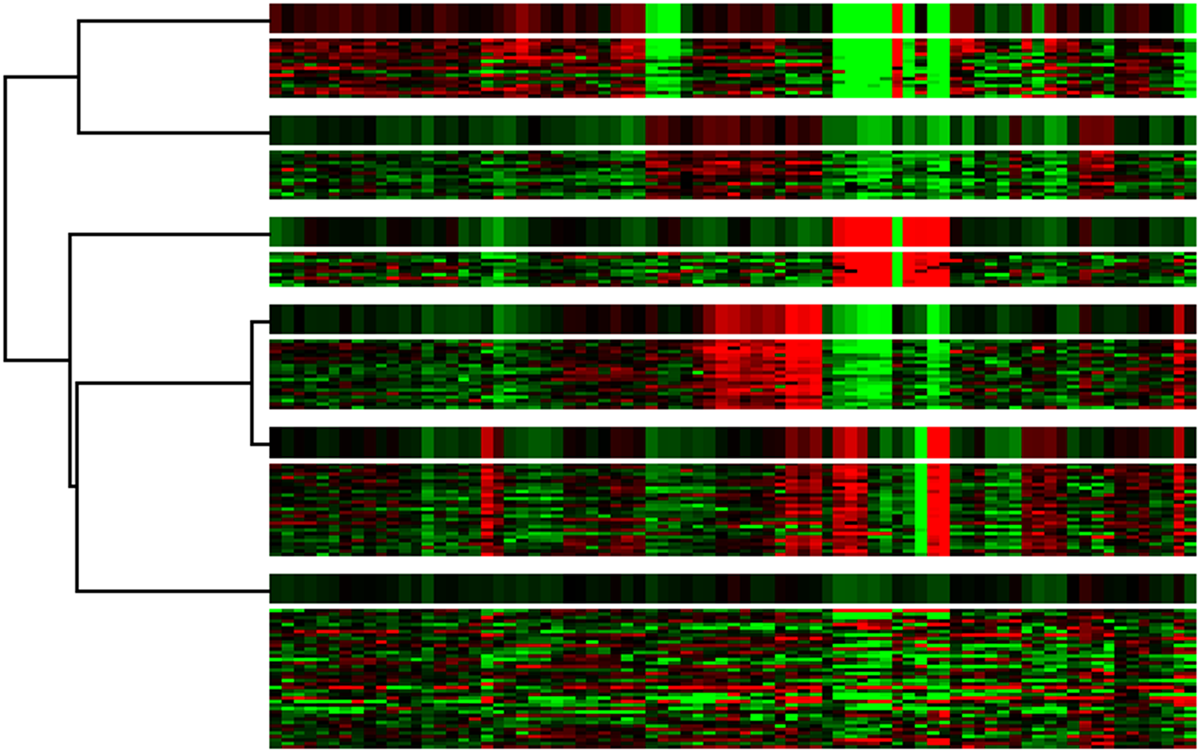
**Dendrogram of averages**. A dendrogram created from cluster averages with the genes in a cluster displayed below each average. The length of each branch of the tree is proportional to the distance between the averages. We create the hierarchy from the cluster averages, which allows us to show high level relationships between clusters generated by arbitrary clustering algorithms. (Data and clusters from [19])

In addition to assessing the quality of clusters produced by an algorithm, it is also important to understand how the clusters and genes in different clusters relate to each other. Clusters with similar overall expression profiles may functionally interact with one another. One method to show high level cluster-to-cluster relationships is to calculate a hierarchical clustering using only the averages of each cluster. We can then hierarchically arrange the cluster averages and display the dendrogram relating the averages to each other (Fig. 6). As this method only creates a hierarchy for the cluster averages, rather than for individual genes as in the case of hierarchical clustering of the entire dataset, it allows us to show cluster relationships for arbitrary clustering algorithms.

However, this dendrogram of averages fails to show the relationships between genes in different clusters. It is important to examine gene-to-gene and gene-to-cluster relationships to assess whether or not genes are included in the most appropriate cluster. In order to view the lower level relationships among genes in clusters we can project high dimensional microarray data into a lower dimensional space such that genes with similar expression profiles are spatially closer to each other than genes with different expression profiles. We use Principal Component Analysis (PCA) to define the axes of a three-dimensional space to project the genes and clusters onto. PCA has been used previously in microarray data analysis for dimensionality reduction to facilitate easier analysis and comparisons [4,20] and to identify patterns of noise [21]. Our method is interactive and navigable which allows users to examine individual genes and view relationships between clusters as they separate out spatially.

**Figure 7:**
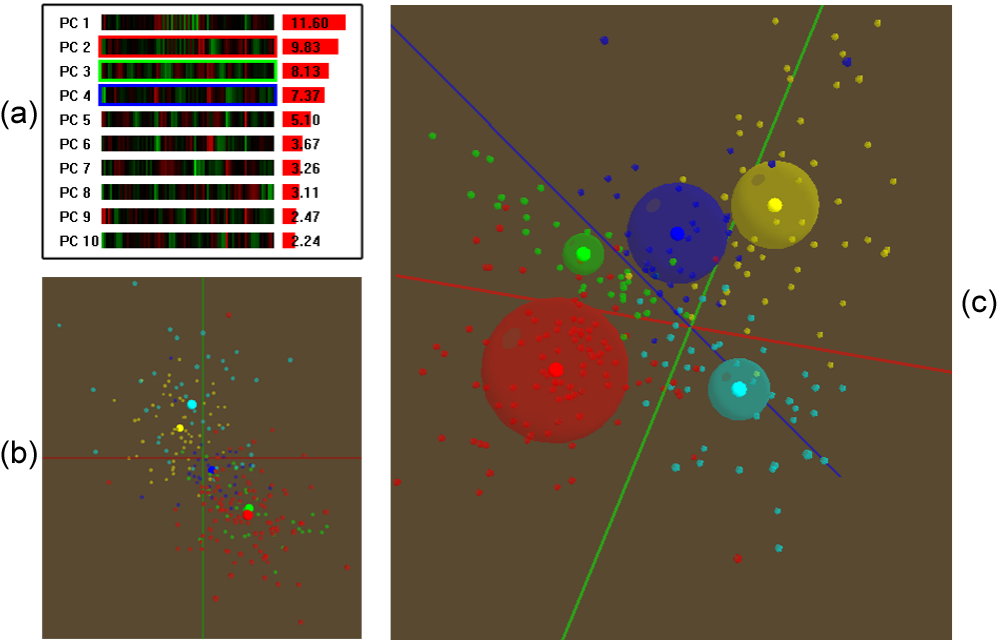
**Principal component projection visualization**. A projection of genes from a cell cycle data set into a 3D space defined by user selected Principal Components. Genes in each cluster are colored by phase (Red-G1, Green-S, Blue-G2, Yellow-M, and Cyan-M/G1). Cluster averages are displayed by larger solid spheres. The much larger transparent spheres show the region included by one standard deviation away from the average. (a) shows the top ten PCs of this data set and the percent of variance accounted for by each PC. (b) is a projection of cell cycle genes onto a space defined by the 1^st ^and 2^nd ^PCs. The separation is poor due to the first PC being highly correlated to noise in this data set. (c) shows the same data projected into a space defined by the 2^nd^, 3^rd^, and 4^th ^PCs. These PCs are highlighted in (a) corresponding to the axis colors in (c). Notice that the cell cycle phases are separated in order around the origin, and that G1 and M phase genes are opposite each other, which is consistent with their opposing expression profiles. (Data and clusters from [22]).

To perform PCA on the microarray datasets, we use Singular Value Decomposition (SVD). SVD decomposes an *m *× *n *matrix of the full microarray data, *X*, into three additional matrices:



Where *M *is the number of genes and corresponds to rows of the matrix, and *N *in the number of experimental conditions and corresponds to the columns of the matrix. We use the eigengenes, or Principal Compenents (PCs), defined in the rows of *V*^*T *^as the axes for our PCA visualization. The position of each gene in that space is determined by the corresponding column of *U*Σ. The square of the singular values, contained on the diagonal of Σ, correspond to the variance included by each PC such that the percent of variation, *p*, captured by the *k*^*th *^PC is determined by:



In this formulation, the singular values are in decreasing order, meaning that the first PC includes more variation than the second, and so on. Thus, using the top 3 PCs includes the most variation possible in a three dimensional projection. We would expect that well-formed clusters would separate out the most when using the top PCs as the axes of projection. However, in some data sets the top PCs are not the most appropriate space for projection. For example, in the Spellman *et al*. cell cycle data set [22] using our tool we can see that the first PC does not show the "banded" pattern typical of ordered cell cycle data, which the second, third, and fourth PCs do display (Fig. 7a). Accordingly, a projection into the first two PCs does not separate out cell cycle regulated genes/clusters spatially (Fig. 7b). This is consistent with previous PCA analysis done by Alter *et al*. [21] which identified the first PC of this data as highly correlated to noise rather than meaningful information. Our method allows the user to dynamically specify which PCs define each axis, which allows exploration of which PCs are most appropriate for analysis and identification of potential noise-correlated patterns in the data. In the case of Spellman *et al*. cell cycle data, we can use the 2^nd^, 3^rd^, and 4^th ^PCs for projection, which leads to much better spatial separation (Fig. 7c). In this projection, we can see that each phase of the cell cycle spatially separates in temporal order around the origin and that the G1 and M phases appear opposite each other, which is consistent with the underlying patterns of expression for cell cycle genes. Our projection of genes and clusters into a space defined by user selected PCs allows the user to view and analyze relationships on both a cluster-to-cluster basis and a gene-to-gene basis.

### Multiple simultaneous views and scaleable architecture

**Figure 8:**
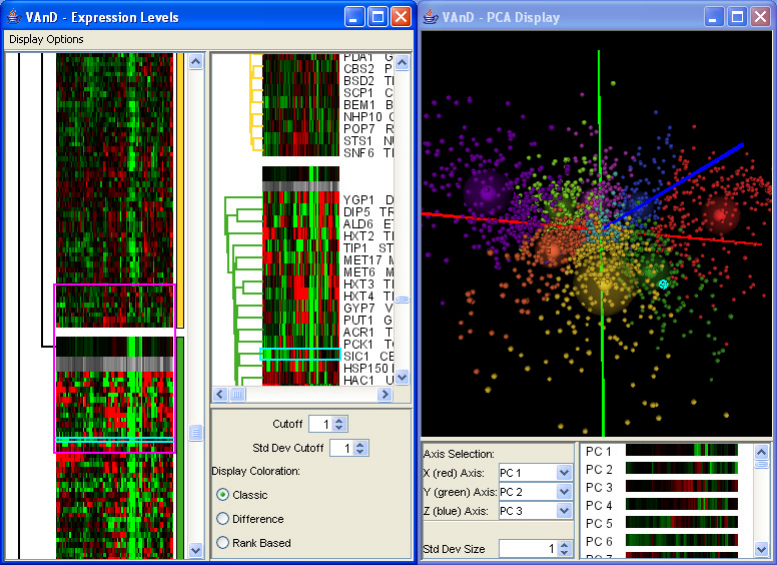
**Multiple simultaneous views**. A screenshot of GeneVAnD displaying clustered data. The panels shown are the expression level window on the left which can toggle between traditional, difference, and rank-based displays, and the PC projection window on the right. A selected gene is highlighted in blue in all views.

In our system each of the visualizations described above are dynamically linked to each other, so that selections, colorations, etc. are shared among views. This allows users to perform tasks in conjunction with one another. For example, using the difference image visualization and the PC projection, users can assess the quality of a clustering as well as the relationship between clusters very easily (Fig. 8).

**Figure 9:**
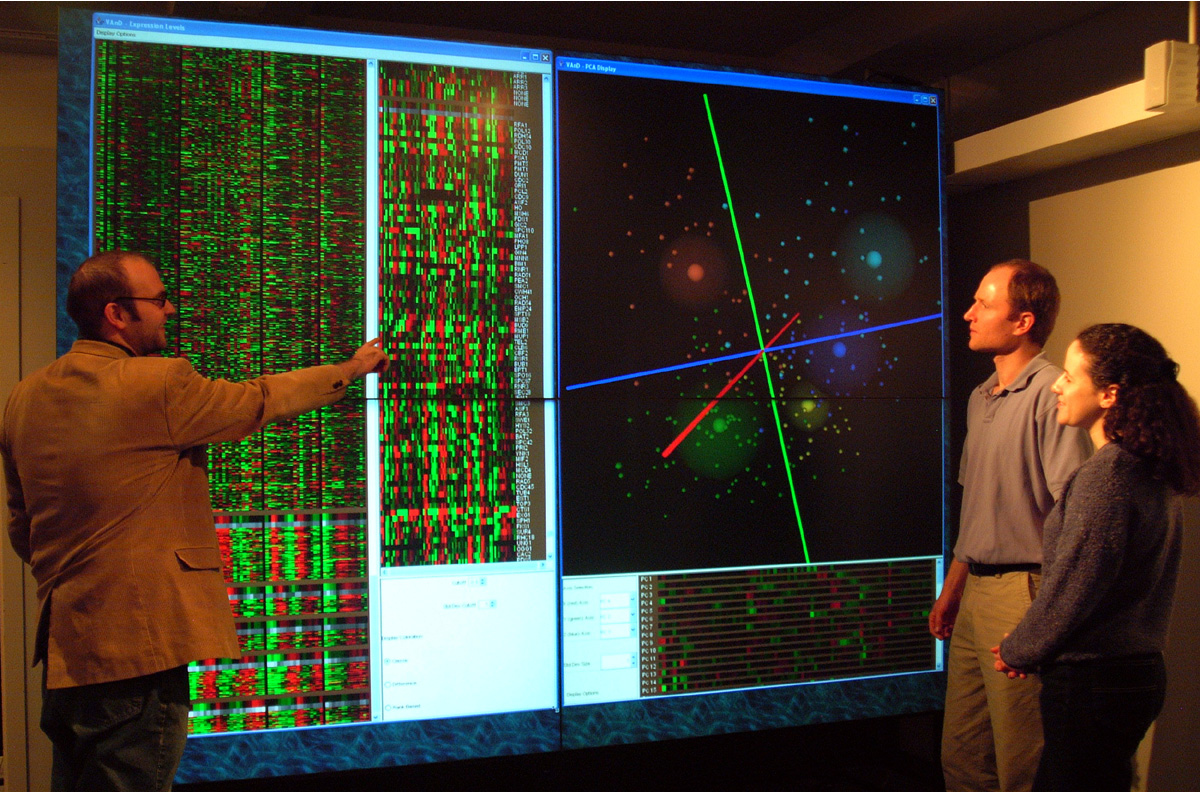
**Large scale display**. GeneVAnD in use on a large-scale display wall. The high resolution enables display of more information simultaneously and the large scale creates an environment conducive for collaboration between multiple researchers.

Our implementation of these methods is both modular and scalable. Although all of the visualizations share a common data structure for dynamic linking, each visualization is displayed in its own panel, allowing for easy addition or removal of new visualization components. Each of the panels is fully scalable for use on both desktop/laptop size displays as well as large display walls. The ability to use these visualizations on large, high-resolution displays facilitates collaboration among researchers and allows users to view greater portions of their datasets simultaneously (Fig. 9).

## Conclusion

Statistical clustering of microarray data is vital for identifying groups of genes that may be functionally related. However the high level of noise in microarray data and the lack of a gold-standard for comparison deeply complicate the evaluation of clustering algorithms. Here we have presented a set of visualization methods geared specifically toward evaluating clustering of microarray datasets. Our rank-based method allows for more noise-robust visualizations of expression levels, our difference display method facilitates visual assessments of general cluster quality as well as outlier detection, and our PC projection method allows for visual assessments of cluster relationships. Our methodology integrates meaningful statistics into an interactive and noise-robust data visualization package for use in analyzing the results of clustering algorithms. Through several examples we have demonstrated the effectiveness of these methods to aid researchers in the analysis of the results of clustering algorithms by facilitating noise-robust assessments of cluster quality and cluster relationships. We believe that more statistically integrative and targeted visualization methods can benefit not only cluster analysis, but many other important data analysis problems in genomics.

## Implementation

Our methodology has been implemented in GeneVAnD (Genomic Visual Analysis of Datasets). GeneVAnD is written in Java and is cross platform for use on Windows, Linux/Unix, and Macintosh operating systems. We use Java3D [23] to display the PC projections and Piccolo [24] to display the expression profiles. The JAva MAtrix Library (JAMA) [25] is used to perform the SVD calculation. The GeneVAnD package is designed in a modular way to allow future extensions and inclusion of additional information and visualizations.

The executables and source code of GeneVAnD can be found at .

## Authors' contributions

MAH and NCD originally conceived the visualization techniques presented and were responsible for initial implementations. MAH created the final implementation of GeneVAnD and drafted the manuscript. KL provided advice and aided in the scalability of the methods to large scale displays and helped draft the manuscript. OGT provided advice and opinions key to the development of the methods and helped draft the manuscript. All authors read and approved the final manuscript.
